# How the relationships between general practitioners and intensivists can be improved: the general practitioners' point of view

**DOI:** 10.1186/cc9061

**Published:** 2010-06-14

**Authors:** Bérengère Etesse, Samir Jaber, Thibault Mura, Marc Leone, Jean-Michel Constantin, Pierre Michelet, Lana Zoric, Xavier Capdevila, François Malavielle, Bernard Allaouchiche, Jean-Christophe Orban, Pascale Fabbro-Peray, Jean-Yves Lefrant

**Affiliations:** 1 Division Anesthésie Réanimation Douleur Urgences, Groupe Hospitalo-Universitaire Caremeau, Centre Hospitalier Universitaire Nîmes, Place du Professeur Robert Debré, 30029 Nîmes Cedex 9, France; 2 Service d'Anesthésie Réanimation B, CHU Saint Eloi, 2 av Emile Bertin Sans, 34000 Montpellier, France; 3 Département d'Information Médicale, Groupe Hospitalo-Universitaire Caremeau, Centre Hospitalier Universitaire Nîmes, Place du Professeur Robert Debré, 30029 Nîmes Cedex 9, France; 4 Service d'Anesthésie et de Réanimation, Hôpital Nord, Chemin des Bourrely, 13915 Marseille Cedex 20, France; 5 Service d'Anesthésie Réanimation, Hôpital Hôtel Dieu, Boulevard Léon Malfreyt, 63000 Clermont Ferrand, France; 6 Réanimation des Urgences, Hôpital Saint Marguerite, 270 Boulevard de Sainte Marguerite 13274 Marseille cedex 9, France; 7 Service d'Anesthésie Réanimation A, CHU Lapeyronie, 371 av Doyen Gaston Giraud, 34000 Montpellier, France; 8 Service d'Anesthésie Réanimation, Service d'Anesthésie Réanimation, Hôpital de la Croix Rousse, 103 Grande Rue de la Croix Rousse, 69317 Lyon cedex 04, France; 9 Service de Réanimation Médico Chirurgicale, Hôpital Saint Roch, 5 rue Pierre Devoluy, 06 006 Nice Cedex, France

## Abstract

**Introduction:**

The present study assessed the opinion of general practitioners (GPs) concerning their relationships with intensivists.

**Methods:**

An anonymous questionnaire was mailed to 7,239 GPs. GPs were asked about their professional activities, postgraduate intensive care unit (ICU) training, the rate of patient admittance to ICUs, and their relationships with intensivists. Relationship assessment was performed by using a graduated visual analogue scale (VAS) ranging from 0 (dissatisfaction) to 100 (satisfaction). A multivariate analysis with stepwise logistic regression was performed to isolate factors explaining dissatisfaction (VAS score, < 25^th ^percentile).

**Results:**

Twenty-two percent of the GPs (1,561) responded. The median satisfaction score was 57 of 100 (interquartile (IQ), 35 to 77]. Five independent factors of dissatisfaction were identified: no information provided to GPs at patient admission (odds ratio (OR) = 2.55 (1.71 to 3.80)); poor quality of family reception in the ICU (OR = 2.06 (1.40 to 3.02)); the ICU's family contact person's identity or function or both is unclear (OR = 1.48 (1.03 to 2.12)), lack of family information (OR = 2.02 (2.48 to 2.75)), and lack of discharge report (OR = 3.39 (1.70 to 6.76)). Three independent factors prevent dissatisfaction: age of GPs ≤45 years (OR = 0.69 (0.51 to 0.94)); the GP is called at patient ICU admission (OR = 0.44 (0.31 to 0.63)); and GP involvement in treatment decisions (OR = 0.17 (0.07 to 0.40)).

**Conclusions:**

Considerable improvement in GP/intensivist relationships can be achieved through increased communication measures.

## Introduction

Because the general practitioner (GP) is a cornerstone of the daily life of the patient and all specialties of the hospital, he or she should be a main communicator with ICU physicians. At patient admission, the GP is the sole medical practitioner who knows the patient's history and his or her way of life. This information could be of particular interest for therapeutic and ethical decisions. In intensive care units (ICUs), GP involvement in the process of family communication is an independent factor of satisfaction among patients' relatives experiencing depression and posttraumatic stress disorder [1-3]. After patient hospital discharge, the sequelae of an ICU stay in a patient's way of life can be severe and prolonged [4,5]. One year after acute respiratory distress syndrome (ARDS), a significant portion of patients have not returned to their previous jobs [4]. For all of these reasons, ICU physicians should optimize their relationships with GPs. However, these relationships are poorly described. In a recent study, 245 intensivists from ICUs in southern France were questioned by phone concerning their relationships with GPs [6]. An informative letter from the GP to the hospital physician was reported for only 20% of admitted patients, and 50% of these letters were considered uninformative. However, only 33% of the intensivists reported contacting designated GPs after patient admission. The lack of informative letters at patient admission and the lack of contact between GPs and intensivists do not reflect good practice. Currently, no study analyzes the relationship between intensivists and GPs at patient discharge. In other fields, Westermann *et al*. [7] reported delayed information by specialists (39 to 46 days after the patient's consultation). Long *et al*. [8] sent a questionnaire to 80 consultants at four hospitals in southeast England and to 100 GPs in the same area. Only 3% of consultants contacted GPs to inform them during the same period. In a postal survey sent from an emergency department to 380 GPs, 147 (39%) responders reported deficiencies in the discharge information and substantial difficulties in accessing outstanding investigation results [9]. In an ENT emergency department, Wasson *et al*. [10] showed that the use of a computerized clinic letter template improves communication with ENT emergency clinic patients' general practitioners. Moreover, adequate communication between emergency departments and GPs (using a referral letter) has been shown to be cost effective, with $2,600 saved per month [11].

Two intuitive reasons could explain the lack of direct contact between GPs and intensivists. First, most patients are transferred to the ICU from another department. Hence, the GPs are not involved in the ICU admission process. Second, the patients are discharged from the ICU to ward, but not to their homes. To increase the collaboration between intensivists and GPs, the present study aimed at assessing the opinions of GPs about their relationships with intensivists.

## Materials and methods

The present study was approved by the Comité Consultatif de Protection des Personnes en matière de Recherche Biomédicale (February 18, 2005, Comité Sud-Méditerranée, n° 05.39). Funding was provided by grants from the French national programme, Projet Hospitalier de Recherche Clinique.

### Design

In this epidemiologic, transversal, descriptive study, an anonymous questionnaire was mailed between June 1 and July 31, 2006, to GPs in four areas of southeastern France (Bouches du Rhône, Hérault, Vaucluse, and Gard). GP addresses were provided by the different French Medical Councils [conseils départementaux de l'ordre des médecins]. In this study, most of GPs were physicians with ≥2 years of residency after the end of their medical studies.

### Questionnaire description

The questionnaire was created and validated by a Survey Committee (five intensivists (BE, SJ, PM, JMC, and JYL) and two epidemiologists (TM and PFP)) and was divided into five parts (English version in Additional file 1).

#### Professional characteristic of GPs

Every GP was asked about his or her gender, age range (25 to 35 years; 36 to 45 years; 46 to 55 years; or > 55 years), first medical school, date of degree certification, onset of professional activity, working area, population of working city, and distance between their office and the nearest university hospital ICU. In addition, information concerning the number of patients per year, the way medical information related to the patient was collected (health book, health card, computer, or none), and continuing medical education sessions was requested.

#### Intensive care training

Every GP was asked about university, postgraduate ICU training including specific areas (cardiac arrest resuscitation; central venous catheter insertion; tracheal intubation; miscellaneous; none; and frequency of the use of ICU abilities).

#### The relationship at admission and during the patient's stay in ICU

Every GP was asked about the rate of patient admission to the ICUs per year, the way they were informed of patient ICU admission (patient's relatives; intensivists; or no information), their communication channels during the ICU stay (visit; phone call; relatives; or hospitalization discharge report), and their contact during ICU visits (intensivist, nurse, or resident). Moreover, every GP was asked about the occurrence of a GP/relative meeting during the patient ICU stay (frequency and the aim of these meetings). The frequency of reception of an ICU report was estimated.

#### GPs' wishes

General practitioners were asked about their wishes concerning the mode of communication at patient ICU admission (letter, phone call, or e-mail) and their level of involvement in treatment decisions.

### Global assessment of the relationship

At the end of the questionnaire, the relationship was assessed by using a graduated visual analogue scale ranging from 0 (dissatisfaction) to 100 (satisfaction).

#### Mailing

The questionnaires were sent with a stamped return envelope to the address of the principal investigator. The questionnaires were sent only once (that is, no reminders) to keep the GP's identity anonymous, despite the risk of decreasing the response rate. A letter written by the president of each Medical Council was joined to the questionnaire to encourage GP response. Responses were collected up to the 31 December, 2006.

#### Data collection

Data were collected by using Microsoft Excel and forwarded to the medical information department of Nîmes University Hospital, Nîmes, France. Statistical analysis was performed by using SAS/STAT 8.1 software (SAS Institute, Cary, NC, USA).

Quantitative variables were expressed as means (± standard deviation) or medians with interquartiles (IQs) according to their distributions. Qualitative variables were expressed as numbers and percentages (total can slightly differ from 100% because of rounding).

General practitioner's dissatisfaction was defined by a global score lower than the first quartile. The populations of the first and remaining quartiles were then compared by univariate analysis with χ^2^, Student *t*, or Wilcoxon tests, as appropriate. When a *P *value was < 0.20, the corresponding parameter was entered into a multivariate analysis with stepwise logistic regression to isolate principal explanatory factors of dissatisfaction. The best model was selected by Wald tests, with a statistical significance < 0.05. Finally, the odds ratios were expressed with a 95% confidence interval (95% CI).

## Results

One thousand five hundred sixty-one (22%) GPs responded. Most of them were men aged 45 years or older. Table 1 shows the professional status of the GPs. Half of the GPs worked in a city with > 20,000 inhabitants. The nearest ICUs were within 25 km of the working city for 90% of GPs, whereas university hospitals were within that range for only 54% of GPs. Sixty-nine percent of GPs used computers for storing patient data, and 68% regularly attended training courses. However, 474 (30%) responders indicated that they had no experience with resuscitation maneuvers.

**Table 1:** Professional status

	Number (%)	Missing data
Seniority since thesis (years) (median, (IQ))	22 (15 to 28)	10
Seniority at work (years) (median, (IQ))	20 (13 to 26)	
Size of the working city		17
< 1,000 inhabitants (*n*, %)	53 (3)	
1,000 to 5,000	343 (23)	
5,000 to 20,000	343 (23)	
20,000 to 50,000	214 (14)	
50,000 to 100,000	102 (7)	
> 100,000	489 (32)	
Distance from the nearest ICU		14
< 10 km	973 (63)	
10 to 25 km	413 (27)	
25 to 50 km	149 (9)	
> 50 km	21 (1)	
Distance from the nearest university hospital		13
< 10 km	536 (35)	
10 to 25 km	289 (19)	
25 to 50 km	333 (22)	
> 50 km	390 (25)	
Number of patients per year		129
< 50	44 (3)	
50 to 100	111 (8)	
100 to 200	135 (9)	
200 to 500	192 (13)	
500 to 1,000	479 (33)	
> 1,000	471 (33)	
Storing medical information		
Computer	1,075 (69)	
Health book	582 (37)	
Health card	74 (50)	
None	16 (1)	
Training courses during university		
and postgraduate studies	1,111 (68)	
Training in ICU procedures		
Cardiac-arrest resuscitation	984 (63)	
Central venous cannulation	361 (23)	
Orotracheal intubation	577 (37)	
None	474 (30)	

ICU, intensive care unit; IQ, interquartile; MD, missing data.

### GP-intensivist relationships at admission and during ICU stay

According to the opinion of 1,097 (70%) GPs, at least two of "their" patients are admitted to the ICU per year. Sixty-five percent of the GPs reported to have had no information at patient admission (Table 2). When the GPs were informed, information sources included the family (72%) and the intensivists (39%). During the patient's stay, GPs collected information by phone. Thirty-one percent of the GPs reported that the discharge letter was the only contact with the ICU team. Ninety-three percent of the GPs reported meeting the family during the patient's ICU stay (more than one meeting for 47%). A lack of information (36%) and the poor quality of information (85%) were the two major reasons for the patient's family to meet the GP.

**Table 2:** Information flow to general practitioners at intensive care unit admission and stay

	Number (%)	Missing data
At patient admission (several possible answers)		
By family	1,121 (72)	
By ICU	610 (39)	
By colleagues	108 (7)	
No information	1,010 (65)	
		
During hospitalization (several possible answers)		
Visiting ICU	273 (17)	
Meeting with relatives	216 (14)	
Phone call	1,042 (67)	
Hospitalization report	480 (31)	
		
Interviewer for visit in ICU		79
Senior/junior	662 (45)	
Only senior	167 (11)	
Nurse	6 (0.4)	
Whoever	647 (47)	
		
Meeting between GPs and relatives		46
Never	118 (8)	
Once	661 (44)	
More than once	736 (49)	
		
Family reasons for meeting (several possible answers)		46
No information	559 (36)	
Incomprehensive information	1,320 (85)	
Bad reception in ICU	236 (15)	
Unknown identity or function of interlocutor	297 (19)	
The family trusts the GP	424 (27)	

ICU, intensive care unit; MD, missing data.

### GP and intensivist relationships at patient discharge

Fifty-nine percent of the GPs (897) reported that they were never involved in the treatment decisions concerning their patients. Only 35 (2%) were contacted for all decisions. Fifty percent of the GPs (758) received a clinical report of the ICU hospitalization for each of their patients. When the report was sent, 88% (1,334) of the GPs claimed to read it entirely.

### Global satisfaction and dissatisfaction factors

By using the visual analogue scale, GP satisfaction with the relationship with intensivists reached a median score of 57 (of 100; IQ, 35 to 77). Therefore, the dissatisfaction was defined as a VAS score < 25^th ^percentile, i.e. ≤35/100. The factors associated with GP dissatisfaction are given in Tables 3 and 4. After logistic regression, five independent factors related to GP dissatisfaction were found: no information sent to GPs at patient ICU admission (OR = 2.55 (1.71 to 3.80)), poor family reception in the ICU (OR = 2.06 (1.40 to 3.02)), the ICU's family-contact person's identity or function or both was unclear (OR = 1.48 (1.03 to 2.12)), lack of information for the family (OR = 2.02 (1.48 to 2.75)), and lack of an ICU report at patient discharge (OR = 3.39 (1.70 to 6.76)). In contrast, three independent factors prevent GP dissatisfaction: GP age younger than 45 years (OR = 0.69 (0.51 to 0.94)), information sent to the GPs by the ICU team at patient admission (OR = 0.44 (0.31 to 0.63)), and involvement of the GPs in treatment decisions (OR = 0.17 (0.07 to 0.40)).

**Table 3:** Factors associated with general practitioner dissatisfaction

	Note, ≤35/100 Number (%)	Note, >35/100 Number (%)	Univariate analysis *P *value	Multivariate analysis Odds ratio (CI, 95%)
Age younger than 45 years	120/379 (32)	428/1,116 (38)	0.001	0.69 (0.51 to 0.94)
				
Intensive care training				
Second cycle	137/380 (36)	448/1,121 (40)	0.18	
Third cycle	124/380 (33)	309/1,121 (28)	0.059	
Never	137/380 (36)	365/1,121 (33)	0.21	
				
Information flow at admission				
Information/family	307/380 (81)	783/1,121 (70)	< 0.001	
Information/ICU	68/380 (18)	532/1,121 (47)	< 0.001	0.44 (0.31 to 0.63)
Information/colleagues	14/380 (4)	89/1,121 (8)	0.005	
No information	322/380 (85)	658/1,121 (60)	< 0.001	2.55 (1.71 to 3.80) (1.71 to 3.80)
				
Information flow during hospitalization				
Visit in ICU	51/380 (13)	213/1,121 (19)	0.014	
Meeting with relatives	66/380 (17)	139/1,121 (12)	0.015	
Phone conversation	241/380 (63)	778/1,121 (70)	0.03	
				
Reasons for meetings between GPs and relatives				
No information	194/380 (51)	351/1,121 (31)	< 0.001	2.02 (1.48 to 2.75)
Bad reception in ICU	99/380 (26)	133/1,121 (12)	<0.001	2.06 (1.40 to 3.02)
Unknown interlocutor	102/380 (27)	192/1,121 (17)	<0.001	1.48 (1.03 to 2.12)
Relatives trust the GP	116/380 (30)	300/1,121 (26)	0.156	

ICU, intensive care unit.

**Table 4:** Other factors associated with general practitioner dissatisfaction

	Note, ≤35/100 Number (%)	Note, > 35/100 Number (%)	Univariate analysis *P *value	Multivariate analysis Odds ratio (CI, 95%)
Reception of hospitalization report			< 0.001	
Each patient	118/378 (31)	622/1,102 (56)		1 (reference)
More than one patient of two	128/378 (34)	137/1,102 (12)		3.02 (2.04 to 4.46)
Fewer than one patient of two	104/378 (28)	316/1,102 (29)		1.43 (0.99 to 2.06)
No patient	28/378 (7)	27/1,102 (2)		3.39 (1.70 to 6.76)
				
Hospitalization report reading			0.117	
Precise/in depth	315/363 (87)	982/1,103 (89)		
Rapid scanning	43/363 (12)	106/1,103 (10)		
Conclusion only	2/363 (0.5)	13/1,103 (1)		
Never	3/363 (1)	2/1,103 (0.2)		
				
Association of GPs with treatment choices/decisions			< 0.001	
Never	292/378 (77)	572/1,101 (52)		1 (reference)
Sometimes	7/378 (2)	159/1,101 (14)		0.17 (0.07 to 0.40)
Rarely	77/378(20)	337/1,101 (31)		0.47 (0.32 to 0.67)
Always	2/378(1)	33/1,101 (3)		0.72 (0.15 to 3.33)

ICU, intensive care unit.

### How GPs would like to improve their relationships with intensivists

The main wishes of GPs concerning their patient's ICU stay were to be informed of patient ICU admission, preferably by a phone call, and to be involved in the treatment decisions (Table 5). They would also like the following items to appear in the ICU report: primary diagnosis, adverse events, treatments, and patient management at discharge.

**Table 5:** General practitioner wishes during patient intensive care unit stays

	Number	%	Missing data
Information at ICU admission			16
Yes	1,460	(95)	
No	32	(2)	
No opinion	53	(3)	
			
Mode of information (several possible answers)			16
Phone call	1,107	(71)	
Mail	565	(36)	
Email	501	(32)	
			
Association with treatment decisions			
Always	116	(7)	
When intensivists consider it useful	897	(57)	
Termination of life-sustaining treatment	229	(15)	
No	342	(22)	
			
GP wishes concerning the hospitalization report			37
Current form	840	(55)	
Summary	684	(45)	
Exhaustive	56	(4)	
			
Hospitalization report contents (several possible answers)			
Reason for admission	1,116	(71)	
Summarized evolution	972	(62)	
Daily precise evolution	134	(9)	
Further survey	920	(59)	
Adverse events	923	(59)	
Duration of hospitalization	589	(38)	
Out treatment	1,042	(67)	
Transfusion information	652	(42)	
Key words	382	(24)	
Primary diagnosis	1,151	(74)	
			
ICU hospitalization report in the patient's health book			53
Yes	1,416	(94)	
No	92	(6)	

## Discussion

In this study reporting the opinions of 1,561 of 7,239 GPs who responded to a questionnaire focused on their relationships with intensivists, GPs blamed intensivists for a lack of information at patient admission and discharge and wished to be involved in treatment decisions.

We sent a questionnaire to 7,239 GPs located in the south of France. No recall was performed to favor the anonymity of the responders. This led to 1,561 responses, corresponding to a response rate of 22%. In comparison, Marshall *et al*. [12] used a recall method and obtained 606 responses of 800 anonymous questionnaires sent (response rate, 76%). In the present study, 25% of responders assessed their relationship with intensivists at a score < 35 of 100. This high rate of dissatisfaction among responders indicates that much effort is required to improve GP/intensivist relationships. Because of the moderate response rate, the findings of the present study could under- or overestimate the real opinion of the entire GP population. We cannot determine whether responders aimed at expressing their special interests or conflicts with ICU practices. This lack of information concerning non responders could obtund the analysis. Moreover, as the questionnaire was anonymous, we cannot assess the impact of the practices of the closest ICU on the GP's assessment. Each ICU's visiting policy, that is, their usual communication route with the GP, could influence both family and GP satisfaction. In addition, the studied population may not be representative of the French GP population. The 1,561 responders correspond to only 1.46% of the 106 697 GPs registered in the French National Medical Registry in 2004 [13]. However, the characteristics of the responders tend to be similar to those of the overall French GP population: 64% of GPs were older than 45 years, and more women GPs were in the young range (≤35 years old, 50%; 36 to 45, 45%; 46 to 55, 27%; and older than 55 years: 12% (data not shown)) [14]. No extrapolation to other European countries can be made because the national organization of each country could alter the role of GPs as regards patient care and the GPs' assessment of their relationships with intensivists. Despite these potential limitations, the present study is the largest one ever focused on this subject.

Despite recommendations favoring GP/specialist relations, few studies have reported the actual relationships between these two caregivers. As concerns information exchange between GPs and specialists, Westermann *et al*. [7] reported a lack of information in GP letters (for example, the primary diagnosis or concern for the patient was missing in nearly half of the letters), as well as delayed responses by specialists (39 to 46 days after patient consultation). In another study, Long *et al*. [8] sent a questionnaire to 80 consultants working for four hospitals in southeast England and to 100 GPs in the same area. Only 2% of the GPs contacted (letter, phone, or visiting) the consultant after patient admission, and only 3% of consultants contacted GPs for communication purposes during the same period. After questioning 21 Danish GPs, Berendsen *et al*. [15] concluded that a closer relationship between GPs and specialists may improve patient management. To our knowledge, GP/intensivist relations have rarely or never been investigated. We recently questioned 245 intensivists in southern French ICUs by phone concerning their relationships with GPs [6]. An admission letter from the GP was reported for only 20% of the ICU patients, whereas only 33% of intensivists reported getting in touch with GPs. The former finding was confirmed in the present study because GPs reported that the ICU team informed them of patient admission in only 39% of cases. This lack of information was independently associated with GP dissatisfaction. A similar conclusion was found concerning the relationship between the emergency department and GPs. Montalto *et al*. [16] reported that the letters sent to GPs after a consultation in the emergency department were not informative enough. A lack of crucial information also was reported by 44% of GPs with regard to the correspondence from emergency departments [9]. The lack of information flow to the GPs of ICU patients could lead to the dissatisfaction of the patients' relatives [1].

In daily clinical practice, the present study demonstrates that GP/intensivist relationships should be improved. According to the wishes of the GPs questioned in this study, the following recommendations can be made: a systematic phone call to GPs at patient ICU admission, continuing improvement of patient relative reception and information flow, the participation of GPs in treatment decisions, especially concerning end-of-life decisions, and conveying information to GPs at patient discharge through a short hospitalization report including the reason for admission, the primary diagnosis, and the treatment. The impact of systematic and complete conveyance of information to the patient's GP remains to be studied. Improving the quality of information flow to patients' relatives decreases the psychological consequences, such as anxiety and/or depression [17]. In the present study, improving the information flow to relatives in the ICU could decrease the psychological impact, with potentially fewer visits to GPs by relatives.

The third point has been well explored, especially regarding end-of-life decisions [1,17,18]. This could be of particular importance in France, as half of families do not want to participate in end-of-life decisions [19]. Moreover, the participation of GPs in treatment decisions, especially concerning end-of-life decisions, could prevent the occurrence of posttraumatic stress disorder in family members because GPs remain close to them after patient discharge and/or death. In this sense, GPs could also act as diagnostic screeners for this syndrome.

The fourth point has been studied in emergency departments. Afilalo *et al*. [20] showed that the use of a standardized community system between family GPs and emergency departments increases the quality of transferred information and improves the GP's perceived patient knowledge and patient management. This kind of information has been shown to be preferred to written letters [10].

However, the efficiency of such practices requires further assessment. The implementation of such practices cannot be envisaged without a close collaboration between GP organizations, Medical Councils, and Hospitals.

## Conclusions

The present study shows that GP/intensivist relationships should be improved. Five independent factors of dissatisfaction were identified: no information provided to GPs at patient admission, poor quality of family reception in the ICU, the ICU's family-contact person's identity and/or function is unclear, lack of family information, and lack of a discharge report. Three independent factors are negatively related to GP dissatisfaction (GP age of 45 years or younger, telephone call to the GP at patient ICU admission, and GP involvement in treatment decisions). In conclusion, following the simple recommendations proposed may improve GP/intensivist relationships. Further studies are required to assess actual improvement in GP/intensivist attitudes, and how such improvement affects patient well-being.

## Key messages

• 25% of general practitioners assessed their relationship with intensivists with a score ≤35 of 100.

• Five independent factors of dissatisfaction were identified: no information provided to GPs at patient admission, poor quality of family reception in the ICU, the ICU's family-contact person's identity and/or function is unclear, lack of family information, and lack of discharge report.

• Three independent factors prevent dissatisfaction: age of GPs 45 years or younger, the GP is called at patient ICU admission, and GP involvement in treatment decisions.

## Abbreviations

ARDS: Acute Respiratory Distress Syndrome; CI: confidence interval; GP: general practitioner; ICU: intensive care unit; IQ: interquartile; OR: odds ratio; VAS: visual analogue scale.

## Competing interests

The authors declare that they have no competing interests.

## Authors' contributions

All authors have made substantial contributions to conception and design (BE, SJ, PM, XC, PFP, JYL) or acquisition of data, or analysis and interpretation of data (BE, TM, FM, PFP, JYL) and/or have been involved in drafting the manuscript or revising it critically for important intellectual content and/or have given final approval of the version to be published (BE, SJ, TM, ML, JMC, PM, LZ, XC, FM, BA, JCO, PFP, JYL).

## Supplementary Material

Additional file 1 **English version of the questionnaire**. Click here for file
