# Supplementary material for: How the relationships between general practitioners and intensivists can be improved: the general practitioners' point of view
Source: Crit Care. 2010 Jun 14;14(3):R112. doi: 10.1186/cc9061 (PMC2911758; doi:10.1186/cc9061)
Supplement: Additional file 1 — English version of the questionnaire. [file cc9061-S1.DOC]

**Questionnaire (English version)**

**Professional status:**

1. **Gender:**  Female  Male

**2. Age range:**

 25-35 years  36-45 years  46-55 year  > 55 years

**3. Initial Medical university:**

 South-east  South-west  West  East

 North  Rhône-Alpes  Ile de France

**3. Thesis year: _________**

**4. Setting up year: __________**

**5. Area in which you practise:**

 Bouches du Rhône  Gard  Hérault  Vaucluse

**6. Size of the town:**

 < 1000 inhabitants  1000 à 5000  5000 à 20000

 20 000 à 50 000  50 000 à 100 000  > 100 000

**7. Distance from a University Teaching Hospital:**

 ≤10 km  11 à 25 km  26 à 50 km  > 50 km

**8. Distance from an intensive care unit (ICU):**

 ≤10 km  11 à 25 km  26 à 50 km  > 50 km

**9. How many patients do you treat and follow per year?**

 < 50  50 à 100  100 à 200

 > 200  > 500  > 1000

**10. How do you keep track of your patients? (one or several responses)**

 Health card  Conputer  Health book  none

**Your experience in intensive care:**

**11. Did you have any intensive care training courses during university and post-university time? (one or several)**

 2^nd^ cycle (Final Honours)  3^rd^ cycle (Postgraduate Studies)  Other  Never

**12. Which of these are your abilities in intensive care? (one or several)**

 Cardiac arrest resucitation  Tracheal intubation

 Central venous catheter cannulation  Miscellaneous  None

**Your patients in ICU :**

**13. How often do you hospitalize patients in ICU? (one only)**

 ≥ Once a week  ≥ Once a fortnight  ≥ Once a month

 ≥ Once a quarter  ≥ Once a year  Never

**14. How do you inquire about a patient’s health? (several responses avalable)**

 through relatives  through a colleague

 through ICU  Sometimes no information

**15. Percentage of patients hospitalized in ICU without your knowledge:** _____________%

**Your information in ICU**

**16. When you’re informed of a hospitalization, how do you get more news? (one or several)**

 Visiting ICU  Calling intensivist

 Meeting relatives  Reading discharge letter

**17. When you’re visiting a patient in ICU, who is your favourite interlocutor ? (only one)**

 Only physician

 Resident or physician

 Nurse

 Whoever, what matters most is to get precise information

**18. During ICU stay, do relatives come and see you to get some information from you or for you to get some from the ICU? (only one)**

 Never  Once during hospitalization  > Once during hospitalization

**19**. **Why do relatives consult you during hospitalization? (one or several)**

 Relatives didn’t have any news from the ICU

 Relatives didn’t understand the information given by the intensivists

 Relatives are not satisfied with the ICU welcome

 Relatives didn’t know the identity or function of the interlocutor

 Relatives trust only you

**20. Do you receive a discharge letter at the end of hospitalization? (one only)**

 For each patient  >One patient in two

 < One patient in two  For no patient

**21. Do you read the discharge letter? (only one)**

 Entirely and thoroughly  Entirely and roughly

 Only the conclusion  Never

**Your wishes for ICU**

**22. Which form do you wish for discharge letters? (one only)**

 Current form; it is up to the intensivist to decide

 Summary, laying stress on the important points (1 or 2 pages maximum)

**23. If “summary”, what would you like it to contain? (one or several)**

 Principal diagnosis  Hospitalization key words

 Hospitalization time  End drug treatment

**24. Would you like to be informed of the admission ofone of your patients? (one only)**

 YES  NO  NO OPINION

**25. If “yes”, how? (one or several)**

 Mail  Phone  Email

**26. Do you think you partake enough in the decisions and therapeutic choices during hospitalization? (one only)**

 Always  Sometimes  Rarely  never

**27. Would you like to partake? (one response only)**

 Yes, always

 Yes, if intensivists think it useful

 Only in case of end of life decision

 No, I have confidence in intensivists

**28. Do you think that ICU hospitalization should be noted in health book ? (one only)**

 YES  NO

**Assessment of your relationship with intensivists:**

**29. Make a vertical line on the scale to assess your relationship with intensivists: On the left = minimal satisfaction – On the right = maximal satisfaction**

**Minimal satisfaction Maximal Satisfaction**
